# A Mixed Application of Geographically Weighted Regression and Unsupervised Classification for Analyzing Latex Yield Variability in Yunnan, China

**DOI:** 10.3390/f8050162

**Published:** 2017-05-11

**Authors:** Oh Seok Kim, Jeffrey B. Nugent, Zhuang-Fang Yi, Joshua P. Newell, Andrew J. Curtis

**Affiliations:** 1 Geography Doctoral Program, University of Southern California, Los Angeles, CA 90089, USA; 2 Department of Economics, University of Southern California, Los Angeles, CA 90089, USA; 3 Kunming Institute of Botany, Chinese Academy of Sciences & World Agroforestry Centre (ICRAF) East and Central Asia, Kunming 650201, China; 4 School of Natural Resources and Environment, University of Michigan, Ann Arbor, MI 48109, USA; 5 Department of Geography, Kent State University, Kent, OH 44242, USA

**Keywords:** agricultural yield, mixed method, geographically weighted regression, iterative self-organizing data analysis technique, rubber plantation, Xishuangbanna, Mekong region

## Abstract

This paper introduces a mixed method approach for analyzing the determinants of natural latex yields and the associated spatial variations and identifying the most suitable regions for producing latex. Geographically Weighted Regressions (GWR) and Iterative Self-Organizing Data Analysis Technique (ISODATA) are jointly applied to the georeferenced data points collected from the rubber plantations in Xishuangbanna (in Yunnan province, south China) and other remotely-sensed spatial data. According to the GWR models, Age of rubber tree, Percent of clay in soil, Elevation, Solar radiation, Population, Distance from road, Distance from stream, Precipitation, and Mean temperature turn out statistically significant, indicating that these are the major determinants shaping latex yields at the prefecture level. However, the signs and magnitudes of the parameter estimates at the aggregate level are different from those at the lower spatial level, and the differences are due to diverse reasons. The ISODATA classifies the landscape into three categories: high, medium, and low potential yields. The map reveals that Mengla County has the majority of land with high potential yield, while Jinghong City and Menghai County show lower potential yield. In short, the mixed method can offer a means of providing greater insights in the prediction of agricultural production.

## 1. Introduction

### 1.1. Background

Many of the primary forests in Asia have been replaced by monocultural rubber plantations, causing their status to become a matter of global concern because of the vital role that primary forest ecosystems play in preserving biodiversity. Having originated from Brazilian rainforest rubber trees (*Hevea brasiliensis*), these plantations produce natural latex that is widely used in products, such as tires, medical gloves, prophylactics, and rubber bands [1]. Globally, the area under rubber cultivation has been growing at an annual rate of 3.8 percent per annum, reaching 9.6 million hectares in 2012 [2]. As of 2009, more than 5 million of these hectares were located in the upland Mekong region of tropical China, Thailand, Vietnam, Laos, Cambodia, and Myanmar [3]. Indeed, nearly 93% of global rubber production takes place in Asia [2], though access to suitable lands is now becoming limited [4].

The associated land conversion, accompanied by intensive fertilizer and pesticide use needed for producing natural latex, has greatly threatened regional ecosystems and biodiversity [5,6]. Rubber trees also draw more heavily on groundwater than does indigenous vegetation during dry seasons, leading to water supply concerns [7]. As such, a comprehensive conservation plan in rubber cultivating areas is badly needed. Also, crucially important is an understanding of the biophysical and socioeconomic factors that affect latex yield. These factors can actually explain or even predict land-use and land-cover changes in the region, so that such findings could be utilized for efficient regional land-use planning [8]. This paper applies and modifies a spatial regression technique to provide greater empirical insights into the many determinants of latex yields, how these vary geographically, and to help identify the most suitable regions for producing natural latex in Xishuagnbanna.

### 1.2. Literature Review

Applying spatial regression models has become common in general for explaining how environmental factors affect vegetation and specifically for predicting agricultural yields and identifying their determinants [9–24]. In particular, Geographically Weighted Regression (GWR) is a technique especially designed to incorporate the spatial complexities resulting from variations in scale and location [25], whereas linear regression models and Ordinary Least Square (OLS) estimations are limited in their ability to deal with such spatial complexities. GWR offers three advantages over OLS.

First, GWR is capable of portraying the spatial structures embedded in the data, so it is helpful for researchers to analyze statistical relationships among variables that may vary over space. Several applications of GWR stress the importance of those advantages, such as in explaining agricultural yield across space [9,16]. Second, GWR shows better goodness-of-fit than OLS, quantified in *R*^2^, adjusted *R*^2^, F statistic, Cross Validation (CV), Akaike Information Criterion (AIC), corrected AIC (AIC_c_), or Bayesian Information Criterion (BIC) [10,11,13–15,17–19,21–24,26–28]. Third, the error terms from GWR reflect less spatial autocorrelation than those of OLS, measured by Moran’s *i* [9,13,14,16,23,28], thereby reducing bias in the parameter estimates. Autocorrelation among the error terms of regression models, of course, introduce bias into the parameter estimates.

GWR is not only useful in and of itself, but also enhances the performance of other statistical methods, such as kriging, when used in tandem. A study by Imran et al. [9], for example, demonstrates how GWR enhances predictive accuracy of sorghum yield mapping and ascertains that GWR kriging yields higher accuracy than ordinary regression kriging. In this study, georeferenced yield data are used as the dependent variable while remotely-sensed environmental spatial layers are applied as the independent variables. A GWR is then fitted to those variables and ordinary kriging is applied to the residuals of the GWR; the kriged residual surface is added to the spatial drifts of parameter estimates of GWR, resulting in more accurate spatial drifts compared to the original spatial drifts. In this way, the mixed application of GWR and kriging enables capturing not only the spatial structure through the independent variables but also the spatial structure embedded in the residuals.

Although the main strength of GWR is the ability to analyze and visualize spatial complexity, the number of output maps can be overwhelming, hindering the dissemination of findings, and implying that GWR may not be able to provide a mechanism for summarizing the entire map series. For this reason, it can be useful to apply a multivariate statistical method so as to compress the numerous output maps into more concise ones, which is a common approach in remote sensing. For instance, Iterative Self-Organizing Data Analysis Technique (ISODATA) is a popular classification method frequently used in the field of remote sensing to classify land-cover, landscape, ecosystem, or climatic region by clustering observations in multi-dimensional space [29–32]. In other words, many spectral bands can be streamlined into, for example, one land-cover map. Such an approach, however, has not been tried in the GWR literature. When a multivariate statistical method, such as principal component analysis, is to be used with GWR, the method is usually applied to the inputs of GWR, not the output, so that it can reduce multicollinearity and improve explanatory power [9,11]. The commonality is to streamline a large amount of information into a smaller amount so that a human brain can better perceive and manage it.

### 1.3. Research Questions

In this paper we apply GWR and ISODATA, an unsupervised classification method, to the case study of Xishuangbanna, Yunnan, China, in order to answer the following research questions:

1. What are the determinants of latex yields at the level of Xishuangbanna prefecture as a whole?
2. How do their impacts vary over space at lower spatial levels within that prefecture?
3. In which parts of Xishuangbanna are potential yields the highest?

If a question can be answered properly, it can provide benefits not only in terms of efficiently managing the regional latex production but also in terms of designing an effective land-use plan for conservation, given that there are many smallholders in Xishuangbanna who frequently plant rubber trees haphazardly. Such a lack of land management results in consuming much land and producing little latex. Specifically, GWR’s global model is employed to answer question (1), and the local model application is used to give answers to question (2), and question (3) is answered by applying ISODATA to the local model’s outcome.

## 2. Data and Methods

### 2.1. Study Area

**Figure 1:**
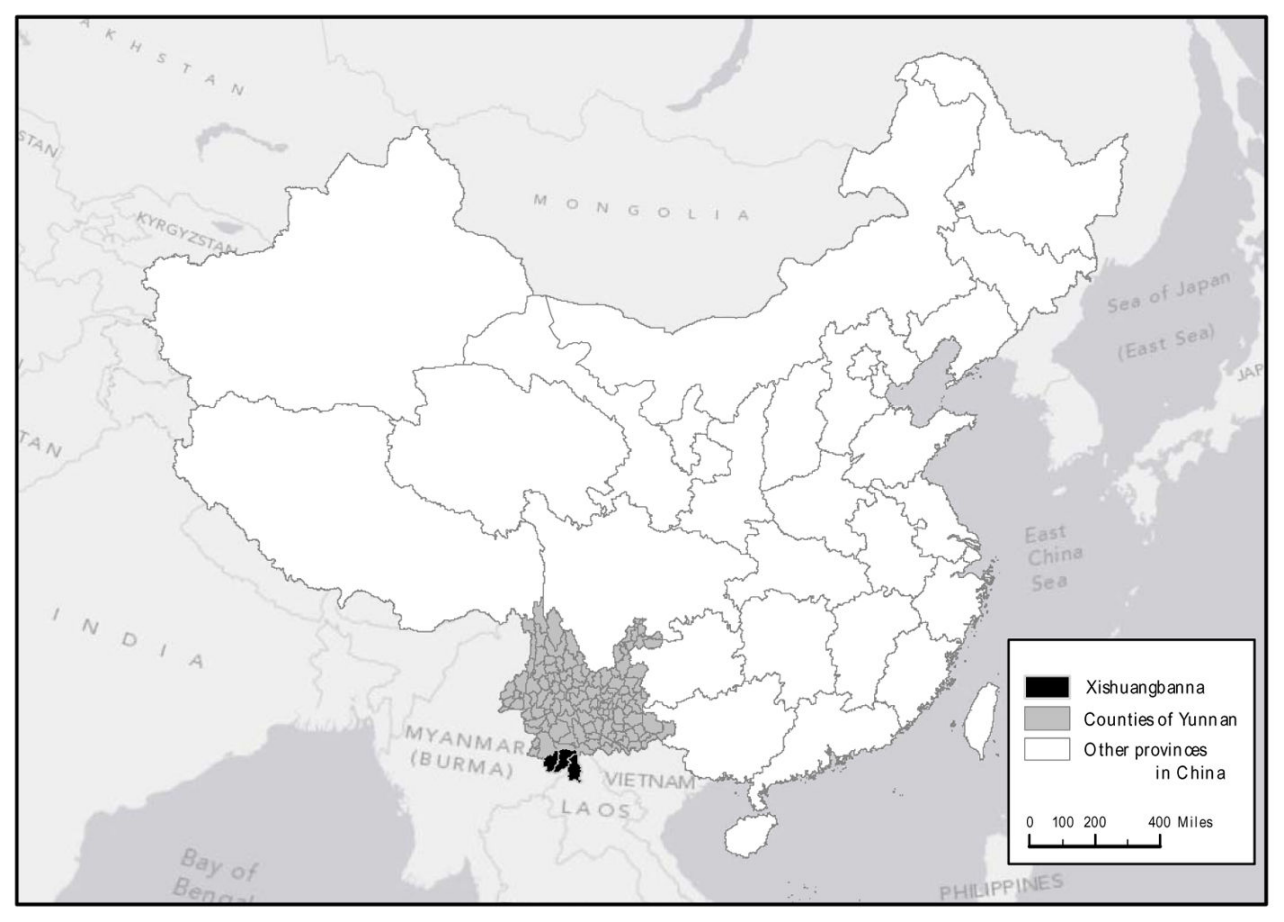
Map of the study area: Xishuangbanna Dai Autonomous Prefecture, Yunnan, China, adapted from Kim [46] with permission.

The Xishuangbanna Dai Autonomous Prefecture (hereafter, Banna) in the province of Yunnan is about 2 million hectares in size, with elevation ranging from 0 m to 1919 m. The latitude and longitude of the southwestern and northeastern corner of the study area is 99.9432° E, 21.1410° N and 101.8382° E, 22.5915° N, respectively. Banna is one of the few tropical areas in China with climatic and geographical conditions quite similar to those of other Southeast Asian countries (Figure 1). At the continental level, it is part of both the Indo-Burma biodiversity hotspot [33] and the Greater Mekong Subregion [34]. Despite existing national land-use policies, Banna remains vulnerable to rapid deforestation and/or forest degradation due to the continuing rapid expansion of the rubber plantations [35–43]. Although the study area was composed almost entirely of tropical rainforest in the past, by 2003 less than half of these forests were left, and only 3.6 percent of that area consisted of old growth tropical rainforests [38,39,44]. This implies a loss of about 6 million tonnes of biomass, or about 14,000 hectares of tropical rainforests, every year since 1976 [45].

### 2.2. Data

**Figure 2:**
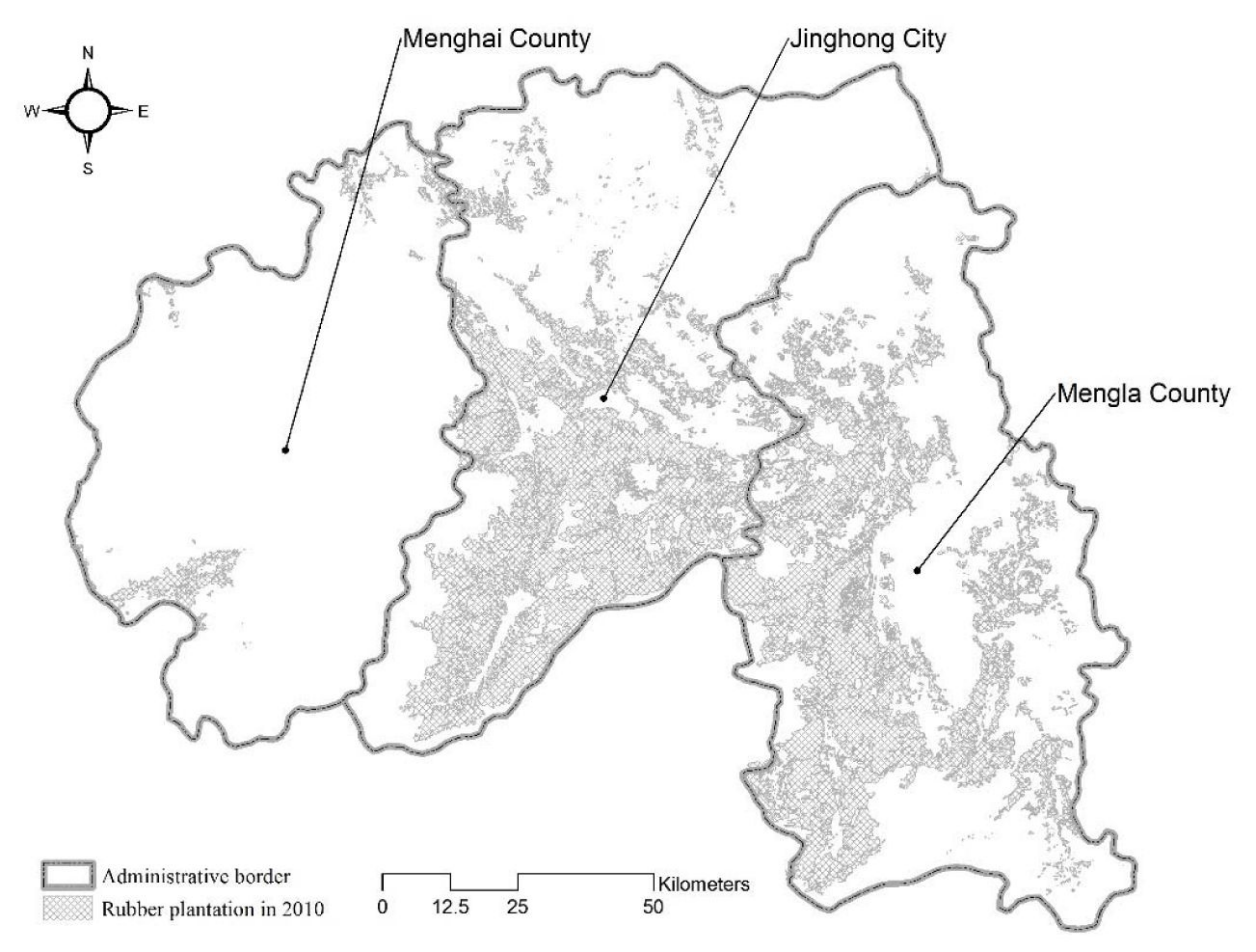
Spatial coverage of rubber plantations in Xishuangbanna (because of a need for data confidentiality, the exact locations of the data points cannot be revealed more precisely in map form. Latex yields and the associated income are closely tied to government subsidies, so to avoid negatively affecting the relationship between the plantation owners and their government, the authors cannot share the information in a spatially explicit form).

Data on yearly latex yields and rubber tree age are constructed from 2008 to 2010 during the wet seasons. The data are physically collected at a plot level, where each plot includes four rubber trees. First, each tree’s daily latex yields and age are measured, so that those measurements can be averaged to represent the corresponding plot. The daily yield data are processed to provide the annual yield. The ages of rubber trees are estimated by measuring length of tapping scars on the barks; approximately 20 cm is equivalent to one year. In all, 1173 plots are selected through stratified sampling of the entire study area, and the data points are georeferenced as points. The shaded areas in Figure 2 identify the spatial coverage of rubber plantations in Banna. (See Yi et al. [7] for more information about the latex yield and age data collection and processing, and Chen et al. [47] for mapping the regional rubber plantations.) The spatial variables included in the modeling are as follows: Percent of clay in soil, the Soil pH from the Chinese Academy of Sciences [7], Annual precipitation from the Tropical Rainfall Measuring Mission (TRMM) [48], Mean, Maximum, and Minimum temperatures from the University of East Anglia’s Climate Research Unit [49], Elevation, Aspect, Slope, and Solar radiation derived from the Global Digital Elevation Map (GDEM) [50], Population from the LandScan [51], Distances from road and stream networks, and Nature reserves from the Chinese Academy of Sciences [7].

### 2.3. Methods

#### 2.3.1. Geographically Weighted Regression

In terms of model structure, GWR is constructed by (1) one global model and (2) potentially many local models. A global model is fitted at the aggregate level whereas the local models consist of a set of similar regressions that are fitted at different but lower spatial scales. The spatial scale indicates the spatial window that is used for running the local models; in other words, the spatial window’s size determines the number of observations used for fitting each regression at the lower spatial scale. Once a spatial window is specified, it will glide over the study area observation by observation. For each move, one regression model is fitted, based only on the observations that fall within the spatial window; numerous regressions are generated because the spatial window will hover around until all of the observations are fully exploited, and these numerous regressions constitute the local model of GWR.

Before fitting a GWR, a Box-Cox transformation is applied to see if any transformation of the dependent variable (i.e., Latex yield) is necessary. The independent variables include the rest of variables, as well as their squares and interaction terms. The squared terms are used for identifying non-linear relationships of where these are deemed relevant. The interaction terms are employed to see whether there are any additional impacts when variables are modeled in tandem. Only those that are statistically significant are retained for the final global model through stepwise selection. These same variables are also used in the local model.

The dependent variable and the residuals in both the global and local models are tested for the existence and variation of any significant first-order spatial autocorrelations using the Moran’s *i* statistics. Similarly, Durbin-Watson statistics are applied to detect first-order serial autocorrelations. Measuring autocorrelation is essential because the data used in this research have both spatial and temporal aspects that might affect the regression outcomes.

The local model includes, in addition to the moving spatial window, a spatial weighting scheme in which observations that are closer to the center of a spatial window are assigned greater weights than those observations that are farther from the center [52]. In other words, the spatial window is characterized by (1) the window size and (2) the distance decay function used for calculating the spatial weights for each observation. The window size can be determined based on either a fixed distance or a fixed number of observations. However, it is the latter “adaptive” spatial window, which automatically adjusts the size of the spatial window based on a fixed number of observations, that is used in this research because configurations of the regional rubber plantations are by nature irregular even though the data points are randomly sampled. A bisquare weights function is used for the spatial weighting scheme to ensure clear local extents for model fitting. Golden section search is used to identify the optimal spatial window, and the values of *R*^2^, adjusted *R*^2^, CV, AIC, AIC_c_, and BIC are applied to evaluate goodness-of-fit. The R ‘spgwr’ package and GWR 4.0 software are used to execute the GWR runs [53,54].

The global model is specified as: $y_{i}=\beta_{0}+\sum_{k=1}^{p}\beta_{k}x_{ik}+\varepsilon_{i}$, and is therefore, locally invariant. The specification of local model is as follows:

(1) $$y_{i}=\beta_{i0}+\sum_{k=1}^{p}\beta_{ik}x_{ik}+\varepsilon_{i}$$

where *y_i_* is the dependent variable at location *i*, *x_ik_* is the *k*^th^ independent variable at location *i*, *β_ik_* is the coefficient for the *k*^th^ independent variable at location *i*, *β_io_* is the intercept at location *i*, and *ε_i_* is the error term at location *i*.

To specify the local model, it is necessary to set an adaptive bisquare weights function *α_ij_*, which is expressed as follows:

(2) $$\alpha_{ij}=\left\{ \begin{array}{ll} {\left(1-\frac{d_{ij}^{2}}{\theta_{i(k)}^{2}}\right)}^{2}, & if\;d_{ij}<\theta_{i(k)}\\ 0, & \mathrm{otherwise} \end{array}\right.$$

where *d_ij_* is the Euclidean distance between observations *i* (denoting the center of the spatial window) and *j* (denoting any other observation within the spatial window);*θ_i_*_(_*_k_*_)_ represents the radius of the adaptive window or the Euclidean distance between observations *i* and *k* (where this adaptive window encompasses the *k* nearest neighbors of observation *i*). Based on this spatial weighting scheme, the weight matrix *W_i_* is constructed as follows:

(3) $$W_{i}=\left[\begin{array}{cccc} \alpha_{i1} & 0 & \ldots & 0\\ 0 & \alpha_{i2} & \ldots & 0\\ \vdots & \vdots & \ddots & \vdots \\ 0 & 0 & \ldots & \alpha_{iN} \end{array}\right]$$

where *N* denotes the total number of observations [55]. Finally, the parameters *β̂_i_* of the local model are estimated as follows:

(4) $${\hat{\beta }}_{i}={\left(X^{T}W_{i}X\right)}^{-1}X^{T}W_{i}ϒ$$

where a nonparametric approach is used to estimate the coefficients. As a result, the local model produces numerous *β̂_i_*s, their values varying over space, and their significance levels indicated by pseudo *t* statistics [52].

#### 2.3.2. Iterative Self-Organizing Data Analysis Technique

Proposed by Ball and Hall [56], ISODATA is a data-driven method for clustering observations in multi-dimensional space. In this research, it is used to cluster the local model’s outcomes into regional strata, so that each can indicate different levels of potential yield to more effectively communicate any findings that might prove valuable for intervention or policy decision-making. When generating a land-cover map from satellite imagery, ISODATA classifies pixels into groups based on spectral similarity. The GWR spatial surfaces can be classified in a similar way. Instead of using spectral bands as variables, one can employ such spatial surfaces for measuring the Age of rubber tree, Percent of clay in soil, Elevation, etc. Each measure identifies a different characteristic of landscape that is meaningful in explaining potential latex yields.

A patch of land (i.e., a pixel) in the study area is characterized by these multi-dimensional spatial surfaces, where each spatial surface of parameter estimates of the local model denotes a single dimension. In multi-dimensional space, ISODATA calculates the cluster means once the number of clusters is specified by the researcher; it then calculates the minimum Euclidean distance between pixels and all cluster means. After the calculation, each pixel is assigned to the cluster that shows the minimum distance; the allocation results in new clusters, and then the cluster means are recalculated. ISODATA repeats such processes until no re-allocation happens or all cluster means stabilize.

## 3. Results

### 3.1. Global Model

Through the Box-Cox transformation, square roots of the dependent variable are considered optimal in fitting the GWR. The final parameter estimates of the global model, based on stepwise selection, are listed in Table 1. The Moran’s *i* statistics of the dependent variable in the global model are 0.74 and 0.47, respectively; they all are significant at the *α* = 0.01 level. From the test results, it is evident that the global model captures spatial structure embedded in the data to some extent. The Durbin-Watson statistic for the global model is 1.88, significant at the *α* = 0.01 level, indicating that it is inconclusive whether or not the residuals retain significant serial autocorrelation.

Table 1 shows that according to the global model, Age of rubber tree has a significant positive relation with Latex yield, which is reasonable—older trees produce more latex than younger ones when other conditions remain the same, but as indicated by the Squared age of rubber tree parameter the older trees end up becoming unproductive at very high age. The lack of significance of the Squared age of rubber tree, however, reflects the observed tendency of both smallholders and local government to replant their rubber trees every 25 and 35 years, respectively [7]. This replanting results in the virtual absence of old, unproductive rubber trees, and this may be the reason why the coefficient of Squared age is not statistically significant in the global model.

Theoretically, the direction of the effect of the Percent of clay in soil on Latex yield could be ambiguous. It could be positive since soil with more clay in it would be expected to hold more water and nutrition than would soil with less clay, but when the Percent of clay in soil approaches 100%, this may make the soil more vulnerable to aridity and hardening, which would negatively affect the Latex yield. As shown in Table 1, however, in the study area its effect turns out to be positive, which is reasonable because few rubber plantations are placed on heavy clay soil. From this outcome, one can learn that clay in Banna facilitates latex production by holding water and nutrition in soil. There is also no evident non-linear relationship for this variable as the squared term is not significant.

The results in Table 1 also show that Elevation is negatively related to Latex yield. The significant and positive parameter estimate of Squared elevation, however, shows that the adverse effect of Elevation on Latex yield eventually fades away and even reverses. Not surprisingly, rubber plantations are mostly planted at medium levels of elevation. Bananas are often cultivated at the lowest areas, while tea or coffee plantations are frequently located at the highest areas [46].

The parameter estimate of Solar radiation is positive, and this too is a reasonable outcome as more solar radiation would result in accelerated tree growth, and hence larger latex yields. Precipitation and Mean temperature are both positively related to Latex yield for the same reason.

Population is negatively related to Latex yield, implying that sparsely populated areas (e.g., rural areas) are showing higher productivity than densely populated areas (e.g., urban areas). The significant and positive parameter estimate of the effect of Squared population indicates that if a rural area is too remote, and hence having a very small size of population, one can expect that the area would grow few rubber trees. The parameter estimate of Distance from road also turns out positive for the same reason.

Distance from stream is also positively related to latex yields. This might seem surprising because it would seem strategic to plant rubber trees close to a water source to assure sufficient water for growing and producing latex. In Banna, however, planting rubber trees near local rivers and streams is strictly forbidden by governmental order to prevent soil loss and conserve fresh water. In other words, Distance from stream in the case study reflects the effect of a deliberate policy intervention, rather than that of the rubber plantations’ physical access to water.

### 3.2. Local Model

Through Golden section search, the adaptive bisquare bandwidth turns out optimal when 508 observations are included for each regression run. The parameter estimates of the local model are summarized in Table 2. The results make clear that the effects of local conditions vary considerably over space and in ways that would otherwise be missed in the global model. All of the parameter estimates range from negative to positive with respect to varying locations, whereas the global model only provides one value for each independent variable.

As can be seen from Table 3, the local model’s goodness-of-fit measure turns out to be better than that of the global model as measured by numerous statistics: larger *R*^2^ and Adjusted *R*^2^, and smaller CV, AIC, AIC_c_, and BIC. The ANOVA test comparing the global and local models indicates that the parameter estimates of local model are significantly non-stationary. As indicated by the Moran’s *i* the local model variant of the GWR application reduces the spatial autocorrelation embedded in the residuals even further—from 0.47 to 0.15.

**Figure 3:**
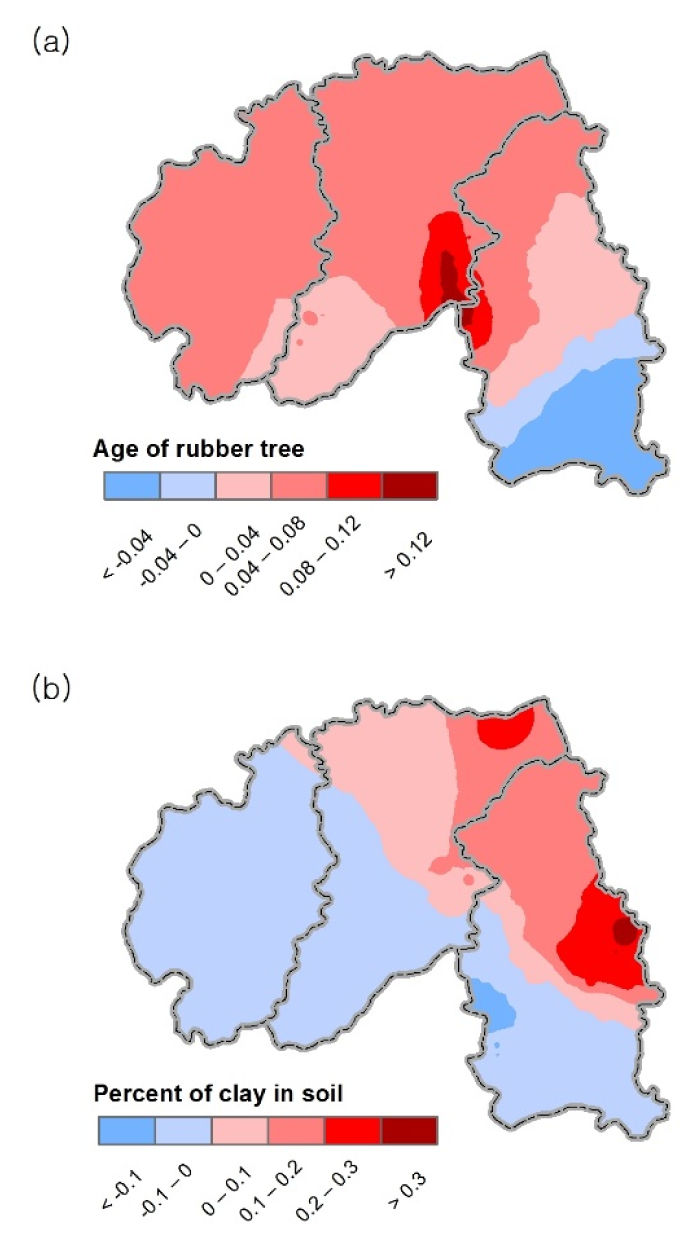

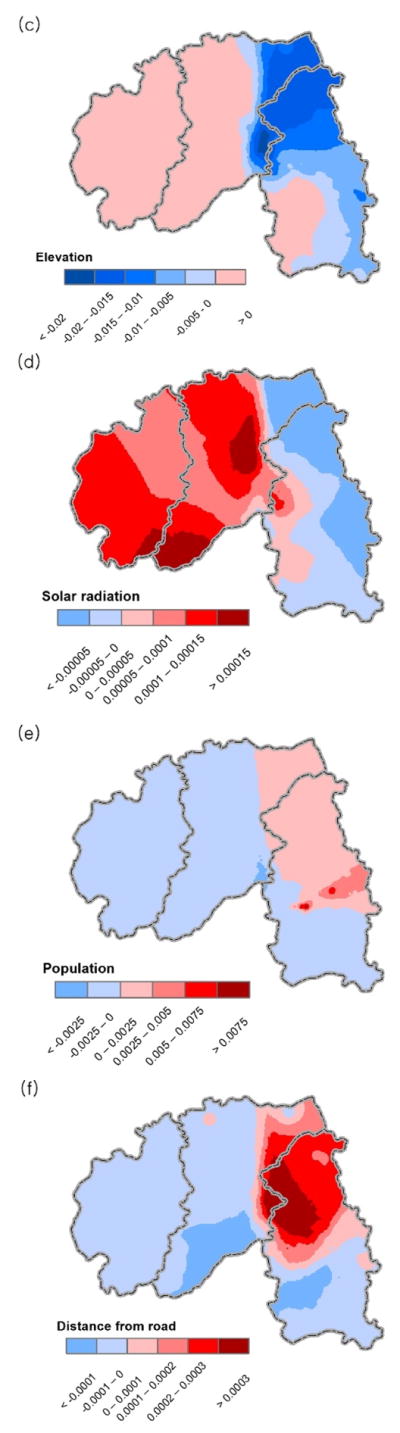

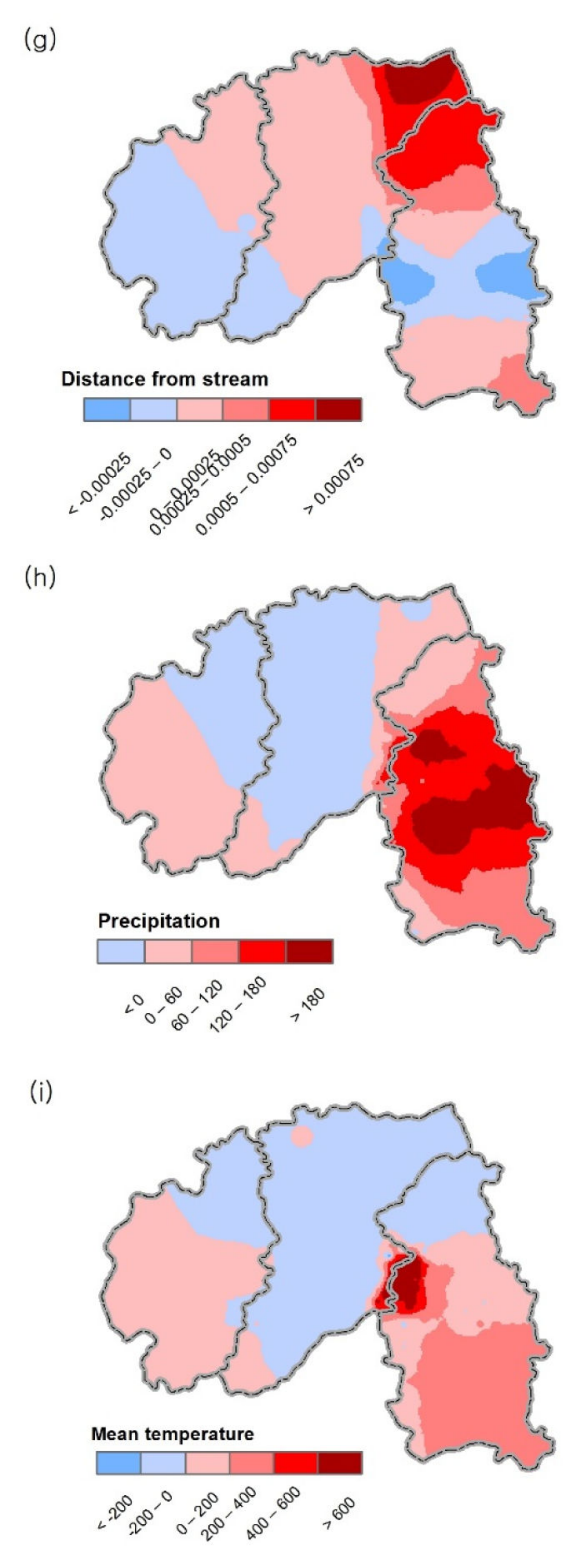
Spatial surfaces of the parameter estimates of (**a**) Age of rubber tree; (**b**) Percent of clay in soil; (**c**) Elevation; (**d**) Solar radiation; (**e**) Population; (**f**) Distance from road; (**g**) Distance from stream; (**h**) Precipitation; and (**i**) Mean temperature, Xishuangbanna prefecture, Yunnan province, south China.

Unlike the global model, the local model provides micro-level, subregional information in a spatially disaggregated yet exploratory fashion (Figure 3a–i). For example, while the global model shows that older trees produce more natural latex than do younger trees when the other conditions stay the same (Table 1), according to the local model, the relationship is reversed in the southeastern part of the study area (i.e., southern part of Mengla), as shown in Figure 3a, i.e., where younger rubber trees are more productive than older ones.

Similarly, although in the global model the Percent of clay in soil is found positively related to Latex yield (Table 1), according to the local model, that positive relation is only valid for the northeastern part of the study area (Figure 3b). There, the clay-rich soil holds more water and nutrition than does soil with less clay, offering conditions that can promote the productivity of natural latex yield. In the rest of the study area, clay-rich soils seem to be vulnerable to aridity and hardening of the soil.

In the case of Elevation, more areas reflect a positive relation between Elevation and Latex yield; however, the areas showing negative relations have larger parameter estimates (Figure 3c). The larger negative parameter estimates for this subregion may explain why the global model indicates that Elevation is on average negatively related to Latex yield (Table 1). The negative relation of Elevation and Latex yield is mainly found in the northeastern and some eastern parts of Banna, and while Elevation is positively related to Latex yield for most of the study area, the actual effect could be minor because the parameter estimates are relatively small in size.

Solar radiation is positively related to Latex yield for most of Banna, and the positive parameter estimates are larger in size than where they are negative as shown in Figure 3d. Yet, Figure 3d also shows that solar radiation is negatively related to Latex yield (as in Table 1). In the eastern part of the study area, solar radiation tends to have an adverse impact on latex production.

More areas show that Population is negatively related to Latex yield (Figure 3e), and the global model also demonstrates the same result (Table 1). In contrast, a sizable positive relation is found in the northeastern part of Banna, implying that more latex yields would be produced with more manpower given that latex yields are manually harvested by farmers every day.

From Figure 3f it can be seen that Distance from road is generally negatively related to Latex yield (in terms of area); however, there are also areas in eastern part of Banna where the parameter estimates are positive. Unlike Population, the positive relation between Distance from road and Latex yield dominates their negative relation (in terms of magnitude), so that the global model indicates that Distance from road is positively related to Latex yield (Table 1). It may be more beneficial to operate rubber plantations in a subregion where roads are closer, and this finding applies to the majority of subregions in the study area.

According to the global model, the parameter estimates of Population and Distance from road are capturing the same information; that is, both variables are regarded as similar proxies differentiating urban from rural areas. This explanation is only valid when it is validated by the local model’s outcomes. In other words, the spatial coverage of Population’s positive parameter estimates has to be similar to that of Distance from road’s negative parameter estimates; otherwise, it is illogical to conclude that Population and Distance from road represent the urban-rural differentiation. According to the local model, however, the two variables do not show that mirrored pattern (Figure 3e, f).

Figure 3g shows that Distance from stream is positively related to Latex yield for most of the study area, a finding consistent with the results of the global model, yet it also shows that in two subregions greater accessibility to streams tends to result in higher latex yields.

Consistent with the results of the global model in Table 1, Figure 3h, i show that Precipitation and Mean temperature are positively related to Latex yield both in terms of areas and the magnitudes of parameter estimates. The exceptions (characterized by negative relations) are found in and around Jinghong City.

Lastly, it should be mentioned that spatial surfaces of the squared terms are not included in this paper since as shown in Kim [57] their outcomes are almost identical compared to the non-squared terms, so that their interpretation would be redundant.

### 3.3. ISODATA

**Figure 4:**
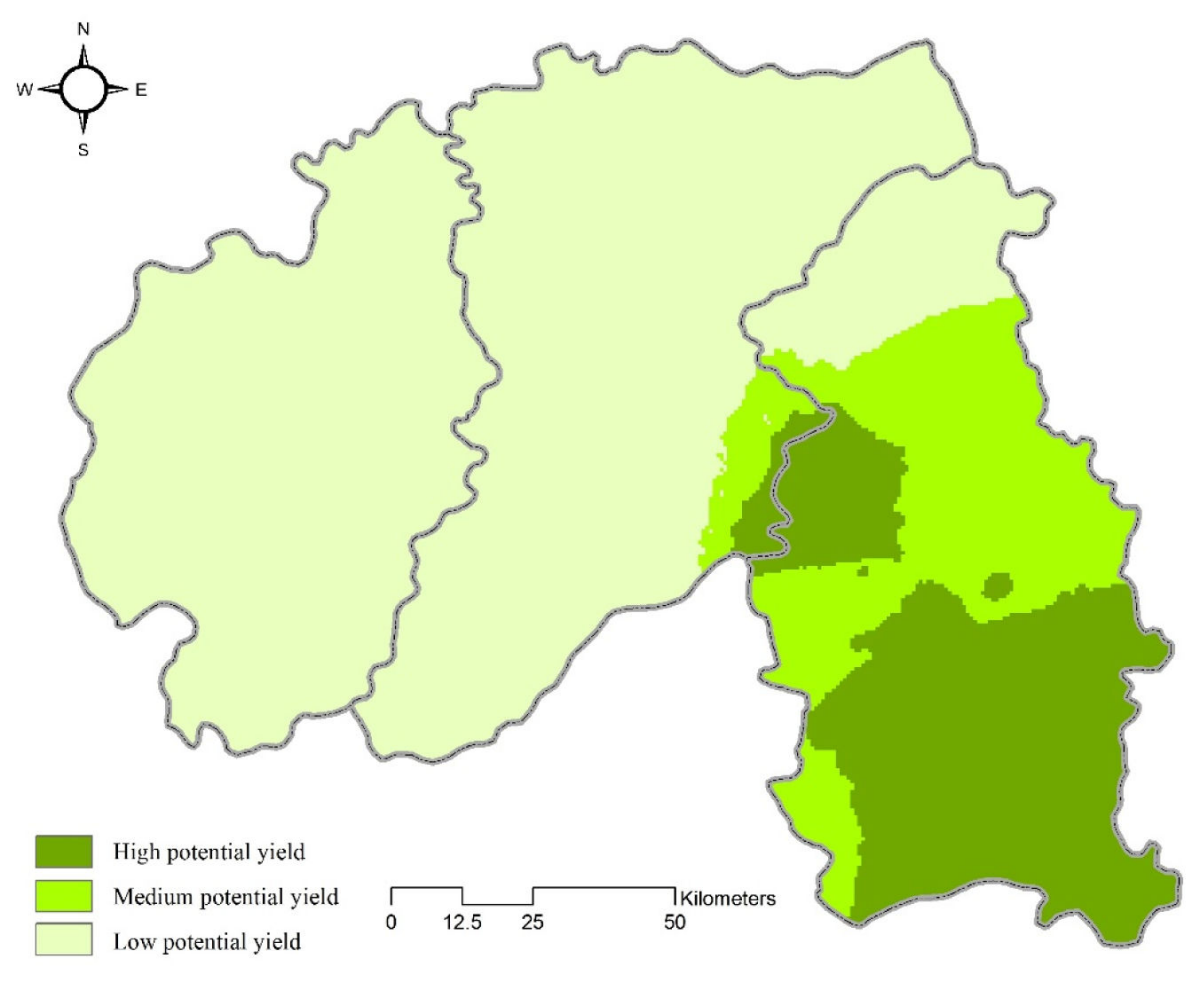
Classified landscape by the mixed application of GWR and ISODATA, showing high, medium, and low potential latex yields, Xishuangbanna prefecture, Yunnan province, south China.

Figure 4 demonstrates the outcome of the ISODATA application on the spatial surfaces. It has been found that three groups are optimal to classify Banna’s landscape in terms of latex potential yield. The dark green indicates the region with the highest potential yield for latex production; the medium and light greens show the areas of medium and low potential yields, respectively. Mengla contains most of the areas with high and medium potential yields. Compared to this fertile land, Jinghong and Menghai are relatively less suitable for producing natural latex (Figure 4).

## 4. Discussion

This paper examines an important ecological problem with inherent spatial complexity. According to the global model, Age of rubber tree, Percent of clay in soil, Elevation, Solar radiation, Population, Distance from road, Distance from stream, Precipitation, and Mean temperature all have statistically significant effects on latex yields, indicating that these are the major determinants shaping latex yields in Banna at the prefecture level. However, the signs and magnitudes of the parameter estimates at the aggregate level are generally quite different from those at the lower spatial levels; their differences have been identified and explanations for them offered. This implies that it is important to interpret the GWR outcomes by considering the global and local models in tandem. From Figure 4 it has been shown that Mengla County, or more generally the eastern part of Banna, is most suitable for producing natural latex, whereas Jinghong City and Menghai County are considerably less suitable. The result, however, does not necessarily mean that Jinghong City and Menghai County are unsuitable for growing rubber trees in an absolute sense.

The map in Figure 4 also displays this usefulness of streamlining many maps into one. Although it loses detail, it facilitates use by the region’s land-use managers as well as the smallholders themselves, and it can facilitate communication between and among them. This is in keeping with a common cartographic concept that map making varies based on the intended audience—the same data being processed to maximize communication to diverse stakeholders.

There are, of course, limitations and uncertainties in our novel exploratory approach as many factors can affect the analysis. Even though the selection of final variables has been systematically carried out through stepwise selection, it is by no means guaranteed that the variable set optimized for the global model would be equally well suited for the local model. One can easily imagine that a variable significant at the aggregate level could be non-significant at a lower (or higher) spatial levels; moreover, not all parameter estimates of the local model are significant. It would be ideal if there is a mechanism similar to the stepwise selection for the local model, but developing and applying that mechanism is beyond the scope of this research. Therefore, it is important to note that there might be some uncertainties due to the imperfect variable selection for different spatial scales. In future research, numerous GWR spatial surfaces should be generated showing the statistical significance of each local model’s parameter estimates so that they could be compared to verify the associated uncertainties. It would be better yet to develop a mechanism to select only the significant parameter estimates of a local model and utilize these for estimating parameters at any location in a dynamic fashion. Wheeler and Tiefelsdorf [58] also proposed similar thoughts for the same issue.

## 5. Conclusions

Basically, our overall analysis is a mixture of producing deterministic outcomes and exploring statistical relationships among variables to find different relationships between latex yields and their determinants across subregions within the Banna region. In other words, the GWR application in this paper considers the global and local models to be complementary to each other. The global model provides helpful outcomes for analyzing macro-level relationships between the natural latex yields and its determinants. By contrast, the local model visualizes spatial surfaces of parameter estimates at a micro-level and helps in finding explanations for any embedded spatial structures in a map form. In sum, although it is exploratory, we believe the proposed mixed method is indeed useful in geographical research and should be regarded as an evolving method. If land allocated to rubber production was in keeping with yield maximization, greater latex production could be obtained with considerably less loss of the natural forests, thereby providing significant environmental benefits.

## Figures and Tables

**Table 1:** Parameter estimates of the global model for latex yields

Variable	Parameter Estimates	*p*-Value
(Intercept)	−273.50	0.00
Age of rubber tree	0.02	0.02
Percent of clay in soil	0.13	0.00
Soil pH		
Elevation	−0.01	0.00
Slope		
Aspect		
Solar radiation	0.00	0.04
Population	−0.00	0.02
Distance from road	0.00	0.00
Distance from stream	0.00	0.10
Nature reserve (dummy)		
Precipitation	35.13	0.00
Mean temperature	30.87	0.00
Maximum temperature		
Minimum temperature		
Squared age of rubber tree		
Squared percent of clay in soil	−0.00	0.00
Squared soil pH		
Squared elevation	0.00	0.00
Squared slope		
Squared aspect		
Squared solar radiation	−0.00	0.10
Squared population	0.00	0.10
Squared distance from road	−0.00	0.00
Squared distance from stream	−0.00	0.06
Squared precipitation		
Squared mean temperature	−0.86	0.00
Squared maximum temperature		
Squared minimum temperature		
Interaction (Percent of clay in soil and Precipitation)		
Interaction (Percent of clay in soil and Distance from stream)		
Interaction (Population and Slope)		
Interaction (Population and Distance from road)		

**Table 2:** Parameter estimates of the local model for latex yields

Variable	Minimum	Mean	Median	Maximum
(Intercept)	−7964.84	−1484.24	−1291.43	2310.32
Age of rubber tree	−0.11	0.03	0.04	0.13
Percent of clay in soil	−0.12	0.02	−0.02	0.32
Elevation	−0.03	−0.00	−0.00	0.01
Solar radiation	−0.00	0.00	0.00	0.00
Population	−0.00	−0.00	−0.00	0.01
Distance from road	−0.00	0.00	−0.00	0.00
Distance from stream	−0.00	0.00	0.00	0.00
Precipitation	−27.89	86.33	89.94	242.54
Mean temperature	−262.04	163.78	139.31	894.24
Squared percent of clay in soil	−0.00	−0.00	0.00	0.00
Squared elevation	−0.00	−0.00	−0.00	0.00
Squared solar radiation	−0.00	0.00	0.00	0.00
Squared population	−0.00	0.00	0.00	0.00
Squared distance from road	−0.00	0.00	0.00	0.00
Squared distance from stream	−0.00	0.00	0.00	0.00
Squared mean temperature	−25.13	−4.53	−3.86	7.47

**Table 3:** Goodness-of-fit and spatial autocorrelation

Statistics	Global Model	Local Model
*R*^2^	0.53	0.76
Adjusted *R*^2^	0.52	0.74
CV	2.48	2.64
AIC	4351.13	3673.84
AIC_c_	4351.13	3686.53
BIC	4442.34	4092.56
Moran’s *i*	0.47	0.15
